# Effects of Glycolysis-Related Genes on Prognosis and the Tumor Microenvironment of Hepatocellular Carcinoma

**DOI:** 10.3389/fphar.2022.895608

**Published:** 2022-07-18

**Authors:** Ju-Yan Zheng, Jun-Yan Liu, Tao Zhu, Chong Liu, Ying Gao, Wen-Ting Dai, Wei Zhuo, Xiao-Yuan Mao, Bai-Mei He, Zhao-Qian Liu

**Affiliations:** ^1^ Department of Clinical Pharmacology, Hunan Key Laboratory of Pharmacogenetics and National Clinical Research Center for Geriatric Disorders, Xiangya Hospital, Central South University, Changsha, China; ^2^ Institute of Clinical Pharmacology, Central South University, Changsha, China; ^3^ Departments of Gerontology, Xiangya Hospital, Central South University, Changsha, China

**Keywords:** hepatocellular carcinoma, glycolysis, prognosis, risk signature, immune infiltrates

## Abstract

**Background:** Hepatocellular carcinoma (HCC) is a common and deadly malignancy worldwide. Current treatment methods for hepatocellular carcinoma have many disadvantages; thus, it is urgent to improve the efficacy of these therapies. Glycolysis is critical in the occurrence and development of tumors. However, survival and prognosis biomarkers related to glycolysis in HCC patients remain to be fully identified.

**Methods:** Glycolysis-related genes (GRGs) were downloaded from “The Molecular Signatures Database” (MSigDB), and the mRNA expression profiles and clinical information of HCC patients were obtained from TCGA. Consensus clustering was performed to classify the HCC patients into two subgroups. We used the least absolute shrinkage and selection operator (LASSO) regression analysis to construct the risk signature model. Kaplan–Meier (K-M) survival analysis was performed to evaluate the prognostic significance of the risk model, and the receiver operating characteristic (ROC) curve analysis was used to evaluate the prediction accuracy. The independent prediction ability of the risk model was validated by univariate and multivariate Cox regression analyses. The differences of immune infiltrates and relevant oncogenic signaling between different risk groups were compared. Finally, biological experiments were performed to explore the functions of screened genes.

**Results:** HCC patients were classified into two subgroups, according to the expression of prognostic-related GRGs. Almost all GRGs categorized in cluster 2 showed upregulated expressions, whereas GRGs in cluster 1 conferred survival advantages. GSEA identified a positive correlation between cluster 2 and the glycolysis process. Ten genes were selected for risk signature construction. Patients were assigned to high-risk and low-risk groups based on the median risk score, and K-M survival analysis indicated that the high-risk group had a shorter survival time. Additionally, the risk gene signature can partially affect immune infiltrates within the HCC microenvironment, and many oncogenic pathways were enriched in the high-risk group, including glycolysis, hypoxia, and DNA repair. Finally, *in vitro* knockdown of ME1 suppressed proliferation, migration, and invasion of hepatocellular carcinoma cells.

**Conclusion:** In our study, we successfully constructed and verified a novel glycolysis-related risk signature for HCC prognosis prediction, which is meaningful for classifying HCC patients and offers potential targets for the treatment of hepatocellular carcinoma.

## Introduction

Liver cancer is the sixth most common malignancy and ranks fourth in terms of mortality in all types of cancer, owing to its highly invasive nature (Bray et al., 2018). The mortality rates, about 2.8% in males and 3.4% in females, increase annually, and patients diagnosed with liver cancer only have 6–20 months of survival period without any intervention (Yang et al., 2019). Hepatocellular carcinoma (HCC) occupies over 90% of liver cancer cases, which is usually accompanied by chronic hepatitis or cirrhosis (Kanwal and Singal, 2019). Due to its hidden and high metastasis, it is rather difficult to accurately diagnose and treat HCC during early occurrence (Imamura et al., 2003). With the continuous development of medical research, the treatment of HCC is also gradually progressed. Curative treatments such as traditional hepatectomy and liver transplantation have been accepted as the main therapies for HCC (Bruix et al., 2016; Zhu et al., 2020); however, since most patients diagnosed are at middle and advanced stages, it is not ideal for them to adopt surgical treatments (Mak et al., 2018). Furthermore, most patients are highly refractory to target therapies such as sorafenib and lenvatinib (Couri and Pillai, 2019). Therefore, the identification of specific diagnostic and prognostic biomarkers for HCC patients is urgently needed.

With the progress of oncology research in the past few decades, energy metabolism reprogramming is regarded as one of the main hallmarks of cancers (Hanahan and Weinberg, 2011). Sufficient energy and biosynthetic metabolic intermediates are the preconditions of cancer cell progression (DeBerardinis et al., 2008). The metabolism pattern of tumor cells was totally different from normal cells; normal cells tend to convert glucose to pyruvate, which was then oxidized to synthesize adenosine triphosphate (ATP) through the tricarboxylic acid (TCA) cycle (Koppenol et al., 2011); normal cells also ferment and consume glucose to lactic acid in hypoxic conditions. However, in cancer cells, even in the presence of abundant oxygen, glucose is predominantly converted into lactic acid accompanied by less ATP production, which is known as the Warburg effect (Wong et al., 2020).

Increasing evidence reveals that high levels of glycolytic flux, with large quantities of glucose consumed and massive amounts of lactate produced, confer advantages to the proliferation, metastasis, and drug resistance of tumor cells (Ganapathy-Kanniappan and Geschwind, 2013; Chakraborty et al., 2017; Bhattacharya and Scimè, 2019). Many glycolysis-related genes are found expressed abnormally and they play a vital role in the development and recurrence of HCC (Zhang et al., 2021). For instance, the glucose transporter type 1 (GLUT1) expression is upregulated significantly in HCC tissues and is positively associated with the tumor size (Sun et al., 2016). Pyruvate kinase M2 (PKM2), a key glycolysis rate-limiting enzyme, is overexpressed in HCC and is a prognosis marker for poor survival; its knockdown inhibits proliferation and metastasis of HCC cells (Li et al., 2020). In addition, elevated PKM2 level is correlated with treatment resistance in HCC patients receiving TACE (Martin et al., 2020). Overexpression of enolase 1 (ENO1), another glycolytic enzyme, in HCC, especially in metastatic lesions, can often indicate worse clinical characterizations including tumor-node-metastasis (TNM) stage, differentiation grade, and poorer prognosis (Jiang et al., 2020). Moreover, hepatic hexokinase 2 (HK2) catalyzes the first step in glucose metabolism; its deletion inhibits tumorigenesis in a mouse hepatocarcinoma model and promotes cell apoptosis (DeWaal et al., 2018). Since aerobic glycolysis plays a vital role in HCC progression; it is of great significance to explore sensitive biomarkers from the perspective of glycolysis to prolong the survival of HCC patients.

In this study, we first screened prognosis-related GRGs *via* analyzing transcriptional data and clinical information in the HCC cohort. Next, using the algorithm and clustering method, we divided the patients into two clusters based on the expression level of screened prognosis-related GRGs. Finally, by means of Lasso regression analysis, we identified a ten-GRG signature, which can effectively predict HCC patients’ prognosis and can be validated in another independent HCC cohort. Our work may lay the foundation for further in-depth studies focusing on the relevance of glycolysis-related genes in HCC and show that glycolytic activity is an important part of personalized treatment of HCC.

## Materials and Methods

### Data Acquisition and Processing

The mRNA expression data and related clinical information of HCC patients were obtained from TCGA data portal (https://portal.gdc.cancer.gov/). TCGA HCC cohort consisting of 374 HCC specimens and 50 adjacent normal specimens were included in this study. The raw transcriptome expression data and clinical information were collated by the Perl programming language (5.32.1.1). Last, only 370 patients with both genomic expression data and clinical data were enrolled for further survival analysis. The detailed clinical information of these patients, including survival status, survival time after diagnosis, gender, age, TNM stage, tumor grade, and pathological stage, is summarized in Table 1. These 370 HCC patients were randomly assigned to the training cohort and the testing cohort at a 1:1 ratio. For further validation, the GSE14520 dataset containing transcription data and survival information of over 200 HCC patients was downloaded from GEO (https://www.ncbi.nlm.nih.gov/geo/).

**TABLE 1:** Clinical pathological features of HCC patients (*n* = 370) from TCGA database.

Characteristic	Group	No. of cases (%)
Age (year)	<65	221 (59.73)
	≥65	149 (40.27)
Gender	Male	249 (67.3)
	Female	121 (32.7)
Survival status	Alive	240 (64.86)
	Dead	130 (35.14)
Pathological stage	Stage I-II	256 (69.19)
	Stage III-IV	90 (24.32)
	Unknown	24 (6.49)
Pathological T	T1-T2	274 (74.05)
	T3-T4	93 (25.14)
	Unknown	3 (8.1)
Pathological N	N0	252 (68.11)
	N1	4 (1.08)
	Unknown	114 (30.81)
Pathological M	M0	266 (71.89)
	M1	4 (1.08)
	Unknown	100 (27.03)
Grade	G1-G2	232 (62.7)
	G3-G4	133 (35.96)
	Unknown	5 (1.34)

### Identification of Glycolysis-Related Genes With Prognostic Significance

To obtain GRGs, we searched the Molecular Signatures Database (MSigDB) using the following terms: “glycolysis” and “glycolytic,” and downloaded four glycolysis-associated gene sets: “HALLMARK_GLYCOLYSIS, KEGG_GLYCOLYSIS_GLUCONEOGENESIS, REACTOME_GLYCOLYSIS and WP_GLYCOLYSIS_AND_GLUCONEOGENESIS” (Liberzon et al., 2015). We deleted the repeated genes from these four gene sets, and 292 GRGs were left. Next, we performed univariate Cox regression analysis to search the prognostic significance of these GRGs in hepatocellular carcinoma patients using the survival package in R. One with *p* < 0.001 was considered a survival-associated GRG. The differential expression of prognostic-related GRGs between HCC and adjacent normal specimens was analyzed using the limma package in R software (Ver4.0.3).

### Functional Enrichment Analysis

To investigate the biological function of prognostic-related GRGs in HCC, the Kyoto Encyclopedia of Genes and Genomes (KEGG) pathway (Kanehisa et al., 2012) and Gene Ontology (GO) enrichment analyses were conducted through the DAVID database (Huang et al., 2009). When performing multiple comparisons, the false discovery rate (FDR) method was utilized to adjust *p* values. The figures were drawn by the ggplot2 package (R software), and pathways with FDR <0.05 were considered significantly enriched.

### Consensus Clustering Based on the Expression of Prognosis-Related Glycolysis-Related Genes

We employed the “ConsensusClusterPlus” R package (Wilkerson and Hayes, 2010) to classify the HCC patients into two subtypes without overlapping. The procedure of clustering was carried out with the iteration number set as 1,000. In each iteration, 80% of the data were sampled, and “k” defined the number of groups. We then performed Kaplan–Meier survival analysis to evaluate whether there is a survival difference between the two clusters by using “survival” R package. Heatmaps showing gene expression differences in the two clusters and the correlation of these clusters with clinicopathological characteristics were generated using the “pheatmap” package. Gene set enrichment analysis (GSEA) was conducted to find whether the glycolysis-associated pathway activity varied significantly between two groups; gene sets were considered significantly enriched when *p* < 0.05 and FDR <0.25 (Thomas et al., 2011).

### Estimation of Immune Cell Infiltration

The stromal and immune scores were measured by ESTIMATE analysis using “estimate” R package, which can calculate the abundance of stromal and immune cells (Yoshihara et al., 2013). A violin plot representing the immune cell abundance between the two clusters was generated by using the “vioplot” package of R.

### Construction and Validation of the Glycolysis-Related Gene–Based Prognostic Risk Score Model

The association between the GRG expression and overall survival of HCC patients was investigated by conducting univariate Cox regression analysis. Prognosis-related genes with *p* < 0.05 were selected, followed by the elimination of highly correlated genes using the “glment” R package (Simon et al., 2011). Ultimately, the optimal value with minimum deviation was calculated by Lasso regression analysis after a 10-fold cross-validation, and, a 10-gene-based risk-predictive model was constructed. The risk score was calculated using the formula: “risk score = β1× Exp (Gene 1) + β2 × Exp (Gene 2) + ⋯ + βn × Exp (Gene n).” HCC patients were grouped into high- and low-risk subgroups based on the median risk score. The KM survival curve analysis and the log-rank test were utilized to estimate the prognosis difference between the two subgroups. ROC curves were utilized to examine the model’s accuracy for prognostic prediction. Moreover, we performed univariate and multivariate analyses to test whether the risk score or clinical features were an independent prognostic indicator. Finally, we employed the CIBERSORT algorithm from the tumor immune estimation resource (TIMER) database to analyze the correlation between the risk score and the infiltration degree of immune cells (Li et al., 2017).

### External Analysis of the Prognostic Signature

To get a better view of the genomic profiles of GRGs, mutation analyses of the GRGs were performed using the cBioPortal database (Gao et al., 2013). We also compared the expression of prognosis-related GRGs at the protein level between normal and HCC tissues by analyzing the immunohistochemistry (IHC) staining images retrieved from the Human Protein Atlas (HPA) online database (Uhlén et al., 2015).

### Cell Culture and Transfection

Human hepatocellular carcinoma cell lines HepG2 and Huh7 were cultured in Dulbecco’s modified Eagle’s medium (DMEM) (Gibco, United States) containing 10% bovine fetal serum (Gibco) and were maintained at 37°C with 5% CO_2_ supply. Transient gene knockdown was achieved by transfecting cells with specific small-interfering RNAs using the Lipofectamine® RNAiMAX Transfection Reagent (Invitrogen, United States).

### Quantitative Real-Time PCR

Total RNAs were extracted from cells using RNAiso Plus (Takara, Japan); then RNAs were converted into cDNA using the PrimeScript™ RT reagent Kit (Takara, Japan). TB Green Premix Ex Taq II reagent (Takara, Japan) was added to the qRT-PCR reaction system according to the manufacture’s instruction. The amplification process was carried out on the LightCycler@480II/96 platform (Roche, Switzerland). Beta-actin was used as an internal control, and the relative gene expression was calculated by the 2^−ΔΔCt^ method.

### Western Blot

Cells were lysed in RIPA lysis buffer (Beyotime, China) for 30 min on ice, followed by centrifugation at 12,000 g at 4°C for 10 min. The supernatant was harvested and protein concentration was measured with BCA Assay Kit (Beyotime, China). Proteins were separated on 10% SDS-PAGE gels and then transferred to PVDF membranes. After blocking in 5% skimmed milk for about 1 h at room temperature, the membranes were incubated with primary antibodies at 4°C overnight. The membranes were visualized in the Bio-Rad gel image analysis system after incubation with corresponding second antibodies on the next day. The following primary antibodies were used in our study: anti-β-actin (A1978, Sigma-Aldrich), anti-HK2 (22029-1-AP, Proteintech), anti-PKM2 (15822-1-AP, Proteintech).

### Colony Formation Assay

Cells were seeded in six-well plates and cultured for about 14 days to allow single clones to form, then the cells were fixed with 4% paraformaldehyde and stained with crystal violet. The colonies were photographed under the microscope. Three independent experiments were performed.

### Migration and Invasion Assay

For the migration assay, 4 × 10^5 cells resuspended with serum-free medium were seeded in the upper chamber of a 24-well transwell culture plate (Corning, United States); then, 600 µl of complete medium was added to the lower chamber. After 48 h, cells that migrated into the lower chamber were fixed, stained, and counted under the microscope. For the invasion assay, the upper chambers were coated with Matrigel (BD Biosciences, United States) before seeding cells. After Matrigel curdled, 8 × 10^5 cells resuspended with serum-free medium were seeded in the upper chamber and 600 µl of complete medium was added into the lower chamber. Migrated cells were fixed, stained, and counted under the microscope 48 h later.

### Statistical Analysis

All data processing and statistical analyses (except for GSEA analysis) were carried out in the R software (version 4.0.3). Differences were considered statistically significant when *p* < 0.05, unless otherwise indicated.

## Results

### Identification of Prognosis-Associated Glycolysis-Related Genes in the TCGA-Hepatocellular Carcinoma Cohort

**FIGURE 1:**
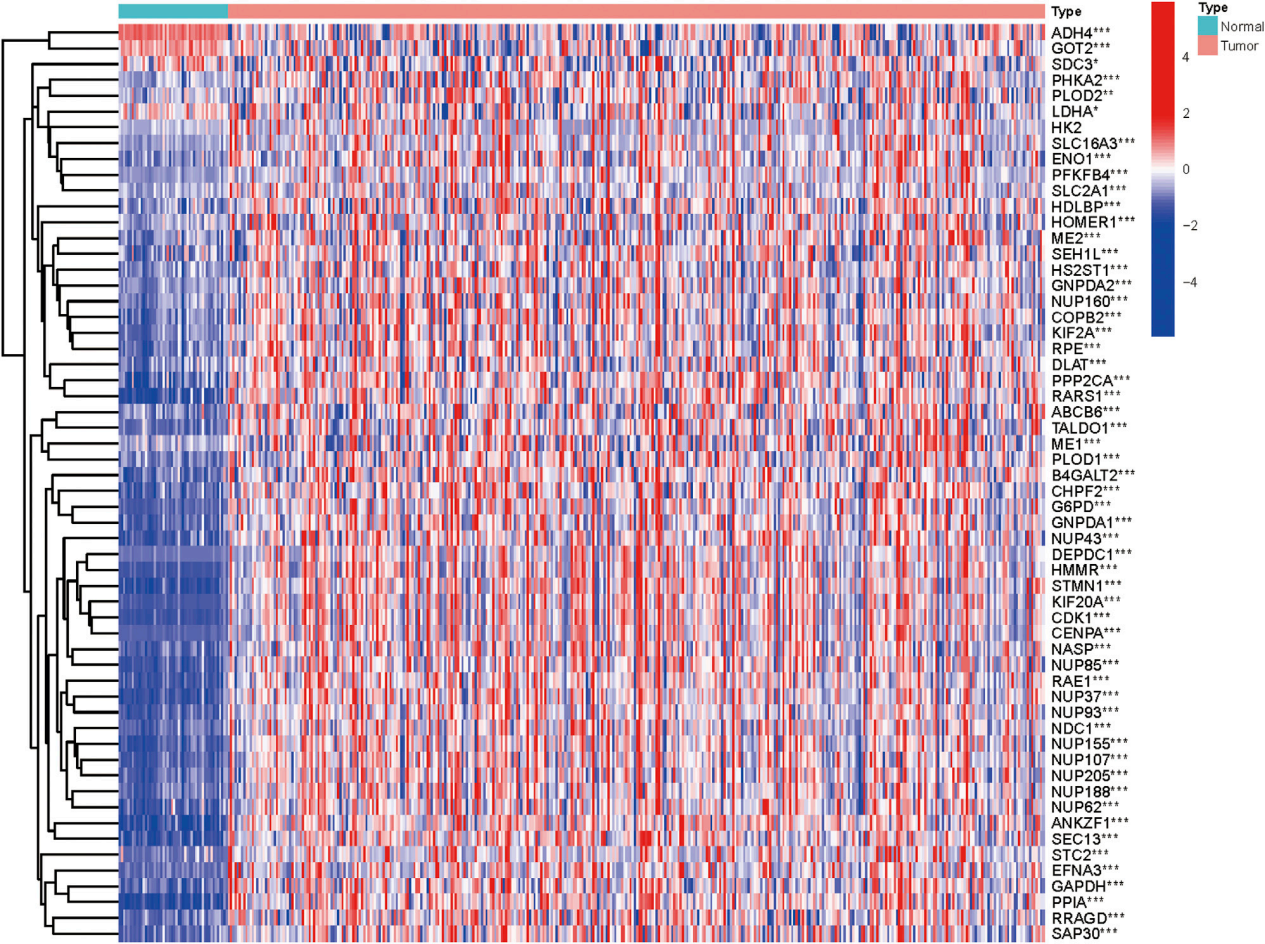
Heatmap showing the mRNA expression of the prognosis-related GRGs between HCC and non-malignant tissues.

We collected clinical data along with expression data of 370 HCC patients from TCGA database. In an attempt to obtain glycolysis-related genes, we downloaded four gene sets from the MSigDB, including “HALLMARK_GLYCOLYSIS, KEGG_GLYCOLYSIS_GLUCONEOGENESIS, REACTOME_GLYCOLYSIS, and WP_GLYCOLYSIS_AND_GLUCONEOGENESIS,” repeated genes were removed and finally 292 GRGs were extracted for analysis. The mRNA expression levels of glycolysis regulators were analyzed, and the differential expression profiles of 292 GRGs between HCC and normal tissues were presented in the heatmap (Supplementary Figure S1). Univariate Cox regression analysis further identified 58 GRGs that were significantly correlated with HCC patients’ overall survival (OS) (*p* < 0.001) (Supplementary Figure S2). The mRNA expression difference of the screened prognostic-associated GRGs between tumor and normal tissues is also indicated in Figure 1. The expression levels of ADH4, GOT2, and SDC3 were obviously higher in normal tissues than in HCC; no significant difference was found for the HK2 expression, whereas others were significantly upregulated in HCC.

**FIGURE 2:**
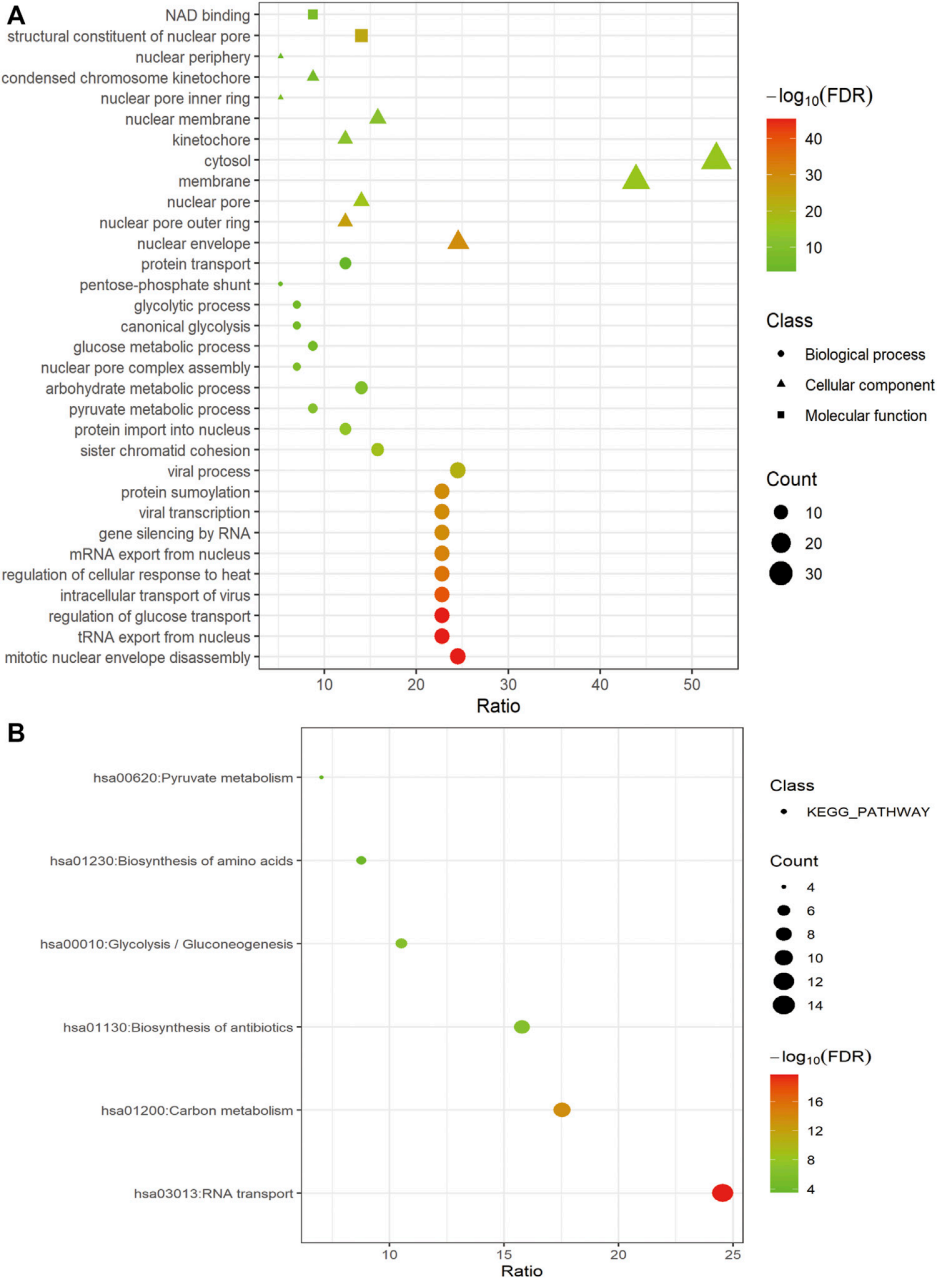
Functional enrichment of prognosis-related GRGs. Bubble charts showing significantly enriched Gene Ontology terms of the biological process (indicated as dots), cellular component (indicated as triangles), and molecular function (indicated as squares) **(A)** and KEGG pathways **(B)**.

For a deeper understanding of the biological functions of the 58 prognostic-related GRGs, we performed GO and KEGG pathway enrichment analysis. GO analysis showed that major pathways enriched in the molecular function part were NAD binding and structural constituent of nuclear pore. The enriched terms of cellular component were “cytosol” and “membrane.” For the biological process, the primary enriched terms were mitotic nuclear envelope disassembly, viral process, tRNA export from nucleus, and so on (Figure 2A). In the KEGG pathway analysis the prognostic-related GRGs were mainly involved in RNA transport, carbon metabolism, biosynthesis of antibiotics, and glycolysis/gluconeogenesis (Figure 2B).

### Consensus Clustering Based on Prognosis-Related Glycolysis-Related Genes Correlated With Clinical Characteristics and Survival of Hepatocellular Carcinoma patients

**FIGURE 3:**
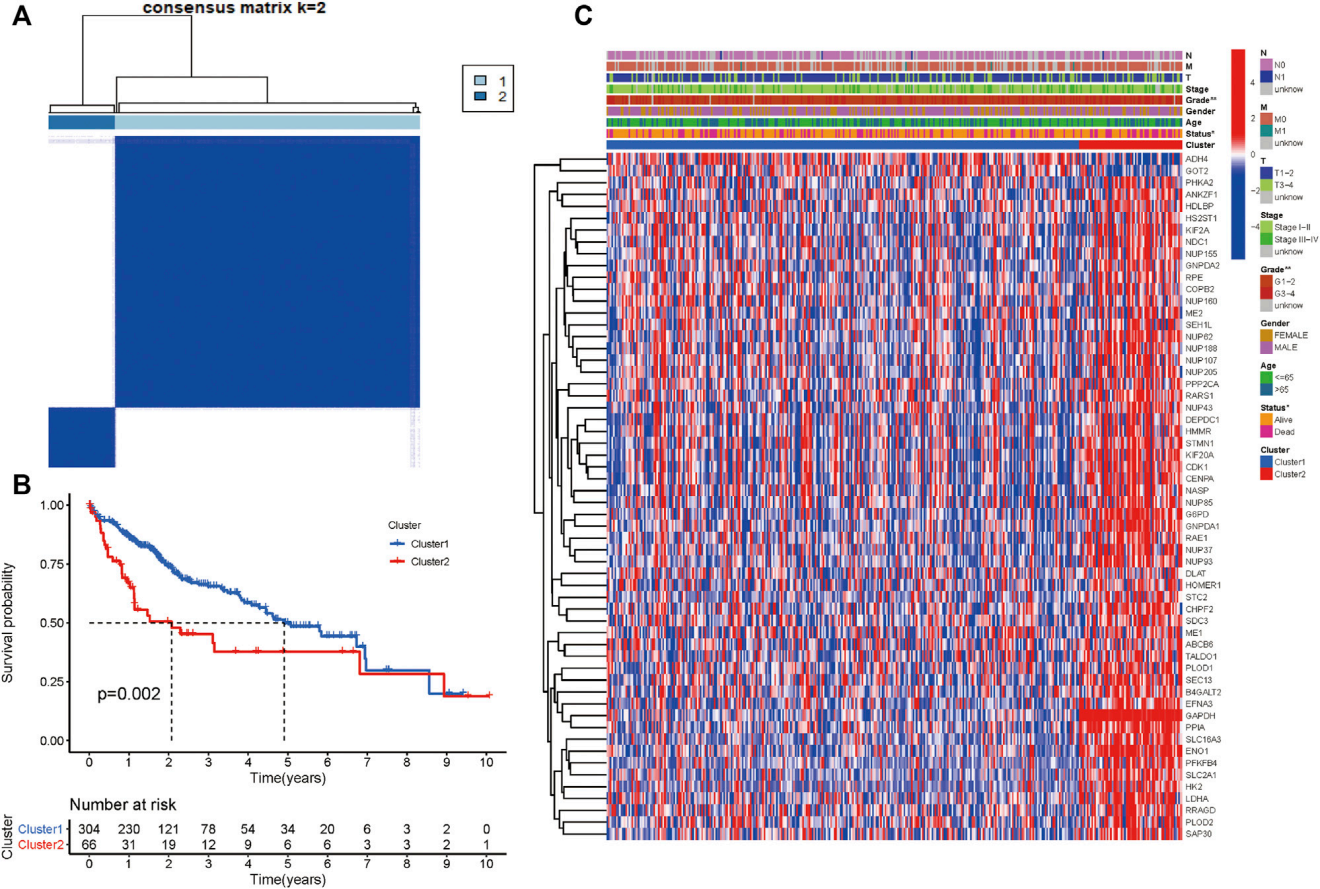
Differential clinicopathological characteristics and survival of HCC patients in two clusters. **(A)** Consensus matrix (k = 2). **(B)** Heatmap showing different clinicopathologic features between the two clusters. **(C)** K-M curves for HCC patients in two clusters.

**FIGURE 4:**
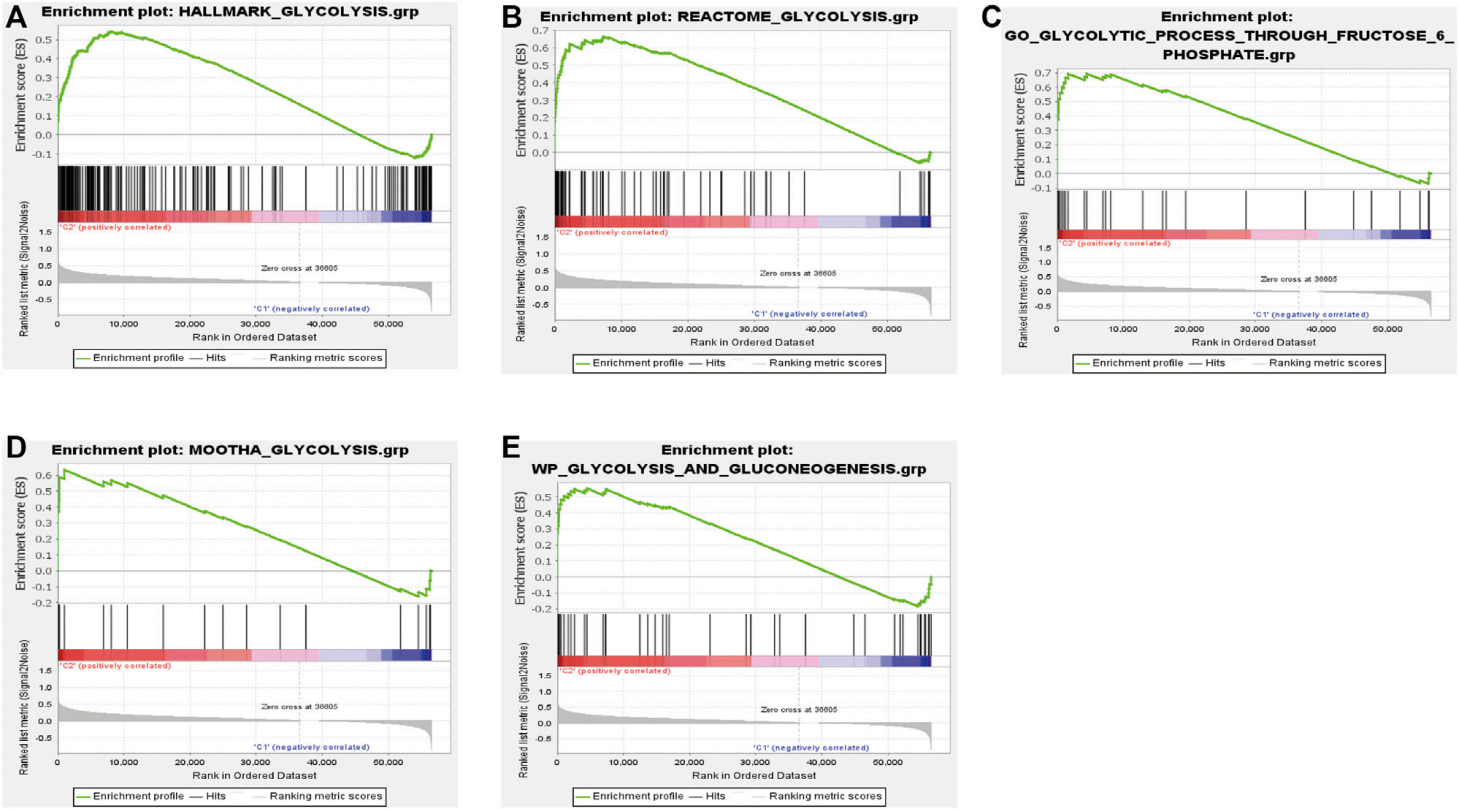
Significantly enriched glycolysis signaling pathways in cluster 2 based on GSEA **(A‐E)**.

We performed consensus clustering to classify 370 HCC patients into different subgroups based on the expressions of the 58 prognostic-related GRGs. When the clustering index k = 2, we obtained the optimal point that indicated the largest differences between two clusters, and there was no significant increase in the area under the CDF curve (Supplementary Figures S3A–C), and almost no overlap was presented between clusters when k = 2 (Figure 3A). Subsequently, a total of 370 HCC patients were divided into two subtypes, with 304 (82.16%) patients in cluster 1 and 66 (17.84%) in cluster 2 (Figure 3A). We then analyzed the expression difference of the prognostic-related GRGs between these two clusters and found elevated expressions of almost all the genes in cluster 2 than in cluster 1, except for ADH4 and GOT2 (Figure 3C). Moreover, K-M survival analysis indicated better OS of patients in cluster 1 than those in cluster 2 (*p* = 0.002) (Figure 3B). The clinicopathological characteristics of the two clusters were also compared; the cluster 1 was closely related to a low WHO grade (*p* < 0.01) and a better survival status (*p* < 0.05) (Figure 3C). Next, we performed gene set enrichment analysis (GSEA) to identify the association of the two clusters with the glycolytic function. The results showed that multiple glycolytic pathways including “HALLMARK_GLYCOLYSIS,” “REACTOME_GLYCOLYSIS,” “GO_GLYCOLYSIS_PROCESS,” “MOOTHA_GLYCOLYSIS,” and “WP_GLYCOLYSIS-AND_GLUCONEOGENESIS” were dynamically correlated with cluster 2 (Figure 4), which is consistent with differential mRNA expression between two clusters. In addition, the violin plot indicated that the abundance of naïve B cells, memory B cells and plasma cells were obviously higher in cluster 1 than in cluster 2, and the stroma score was also higher in cluster 2 (*p* = 0.011), whereas no significant difference of immune score was observed between two clusters (Supplementary Figures S4A–C).

### Construction of a Prognostic Risk Signature Based on the Expression Level of Glycolysis-Related Genes

**FIGURE 5:**
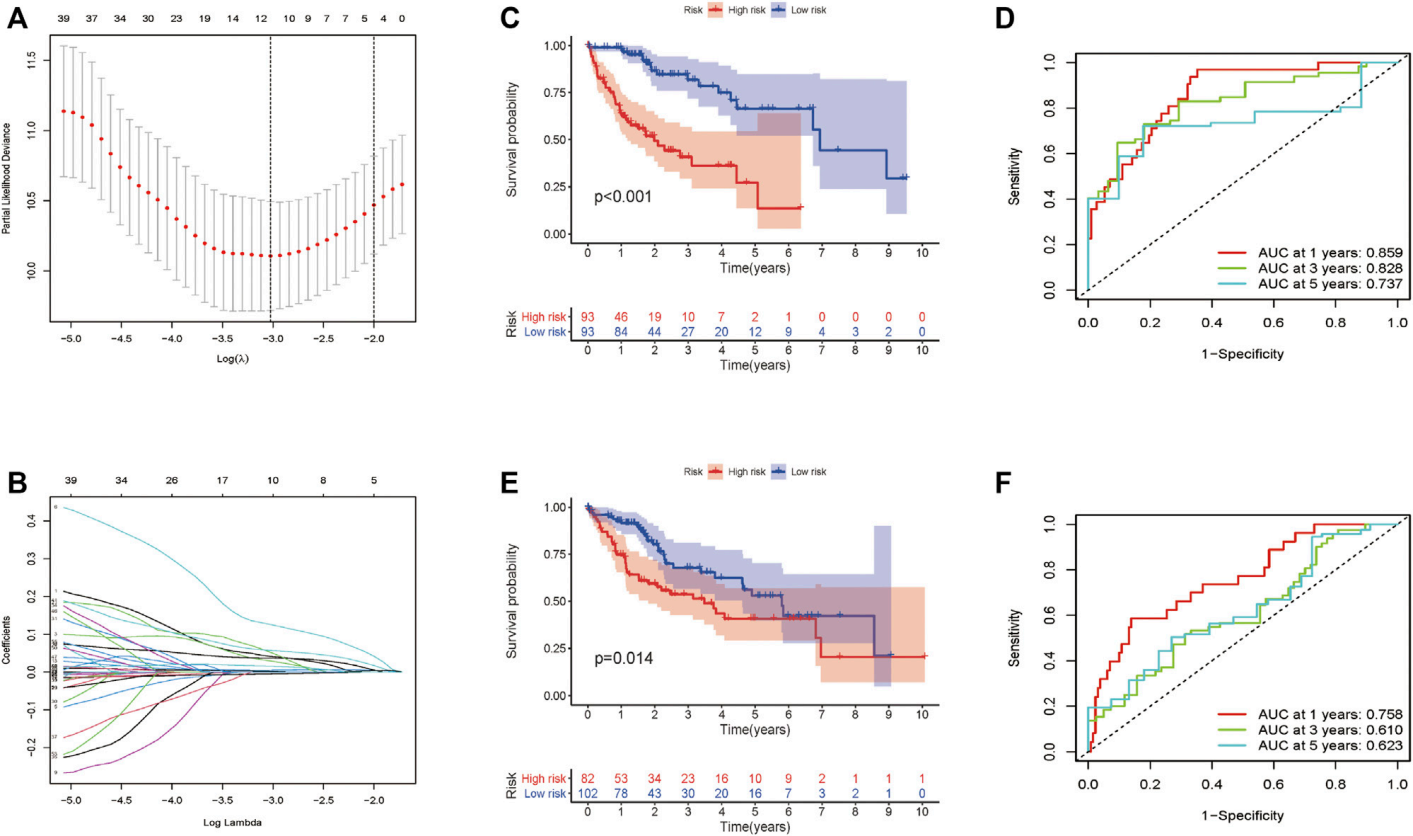
Construction and validation of the prognostic signature for HCC patients. **(A–B)** Lasso Cox regression identified 10 prognostic genes. Survival analysis results in the training cohort **(C)** and the validation cohort **(E)**. ROC curve showing the prediction accuracy of the prognostic signature in the training cohort **(D)** and the validation cohort **(F)**.

**FIGURE 6:**
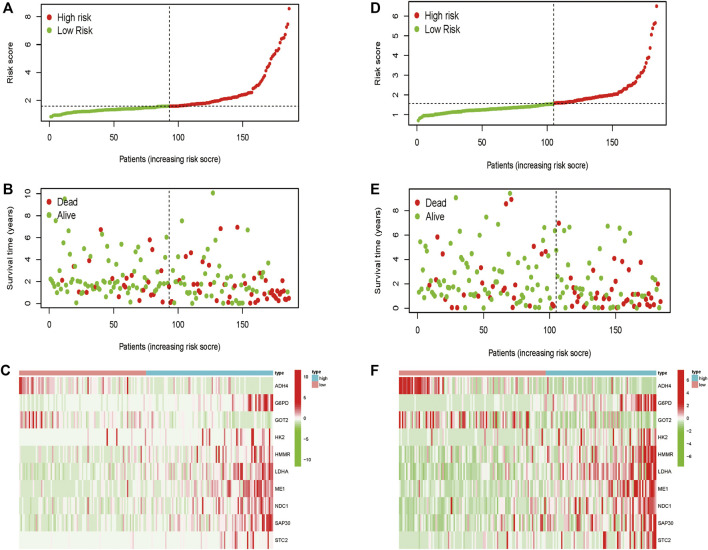
Distribution of the risk score, overall survival, and gene expressions in the training cohort **(A–C)** and the validation cohort **(D–F)**.

Totally 370 TCGA HCC patients were equally assigned to the training group (*n* = 185) and the testing group (*n* = 185) to demonstrate the clinical effects of glycolysis regulators in HCC patients. We further conducted the LASSO regression analysis on the basis of the prognostic-related GRGs screened by univariate Cox regression previously (Figures 5A,B). Ultimately, ten optimal genes (ABCB6, ANKZF1, CENPA, DLAT, G6PD, GOT2, HOMER1, ME1, PHKA2, and STC2) were selected for establishing the prognostic risk model for HCC patients; we calculated the risk score of each patients in the two cohorts with the assistance of coefficients obtained from the LASSO analysis according to the following equation: “risk score = (0.0413*ABCB6 expression level) + (0.029*ANKZF1 expression level) + (0.1258*CENPA expression level) + (0.0367*DLAT expression level) + (0.005*G6PD expression level) + (0.052*HOMER1 expression level) − (0.005*GOT2 expression level) + (0.002*ME1 expression level) + (0.057*PHKA2 expression level) + (0.015*STC2 expression level).” To verify the prognostic ability of the ten-risk gene signature, we divided the patients into low- and high-risk groups based on the median risk score. Survival analysis showed that patients with low-risk scores had better overall survival than patients with high-risk scores both in the training cohort (*p* < 0.001) and the testing cohort (*p* = 0.014) (Figures 5C,E). We also conducted ROC curve analyses to evaluate the predictive accuracy of our identified gene signature. The AUC values at 1-, 3-, and 5 year were 0.859, 0.828, and 0.737 in the training cohort and 0.758, 0.610, and 0.623 in the validation cohort (Figures 5D,F). Moreover, the distribution of the risk scores, survival status, and expression profiles of the ten GRGs-based signatures in two cohorts is shown in Figures 6A–F. To further validate the prognostic efficacy of our risk model in another independent HCC cohort, we analyzed the transcriptional data of GSE14520 dataset and calculated the risk score of each patient. Consistently, K-M survival analysis of the 221 patients with survival information showed that patients with high-risk scores had poorer overall survival (Supplementay Figure S5A). The AUC values at 1-, 3-, and 5 year were 0.573, 0.587, and 0.591 (Supplementary Figure S5B).

### Risk Model as an Independent Prognostic Indicator for Hepatocellular Carcinoma Patients

**FIGURE 7:**
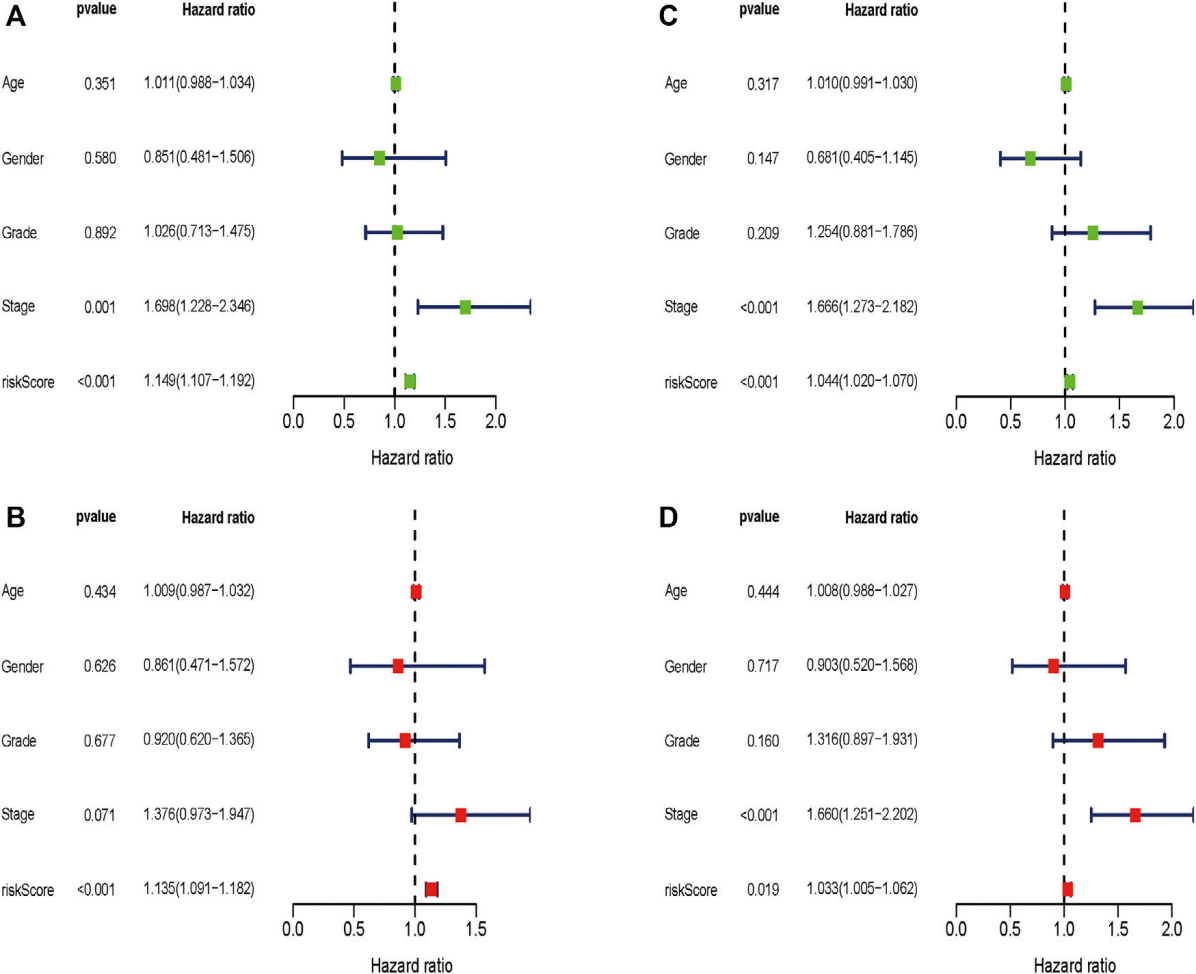
Univariate and multivariate Cox regression analyses in the two cohorts. Multivariate Cox regression analyses in the training cohort **(A)** and the validation cohort **(C)**. Univariate Cox regression analyses in the training cohort **(B)** and the validation cohort **(D)**.

**FIGURE 8:**
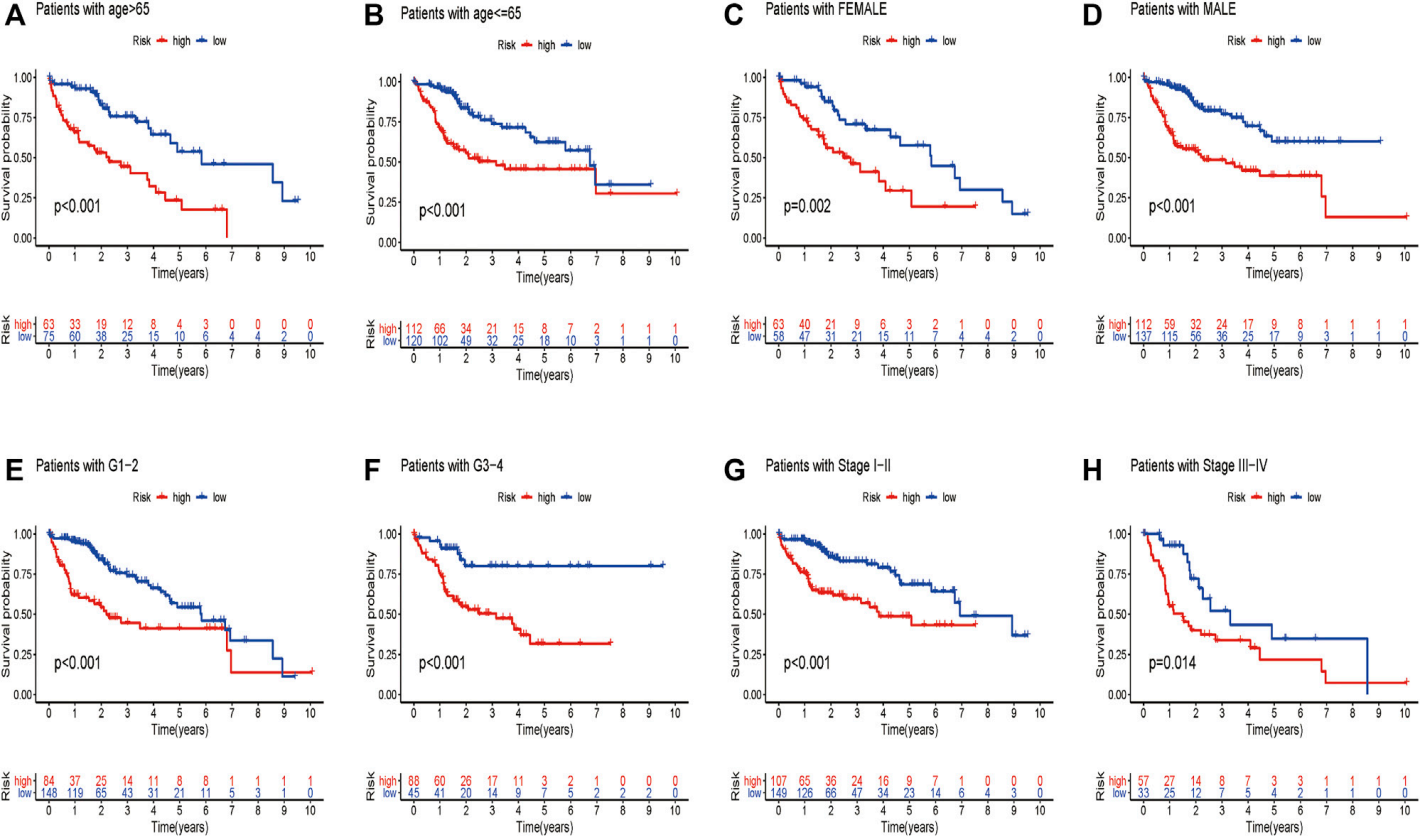
Different overall survival of HCC patients between two groups are shown by classifying patients with age **(A,B)**, gender **(C,D)**, grade **(E,F)**, and stage **(G,H)**.

We wondered if the prediction ability of the risk model could indicate prognosis independently. The calculated risk score and clinical features such as age, gender, tumor stage, and grade were enrolled for our Cox regression analysis. We eventually chose 350 cases for analysis after removing objects with missed clinical information. Univariate analysis showed that the ten-gene risk score and stage were significantly associated with shorter OS of HCC patients in both the training cohort (*p* ≤ 0.001) and the validation cohort (*p* < 0.001) (Figures 7A,C). Next, we explored whether the ten-gene risk score could serve as an independent indicator for the survival of HCC patients, so we additionally performed multivariate Cox regression analyses. In the training cohort, the risk score was independently correlated with shorter overall survival as expected (HR = 1.135, 95% CI: 1.091–1.182, *p* < 0.001) (Figure 7B); similarly, the risk score could also independently predict the prognostic status in the validation cohort (HR = 1.033; 95% CI:1.005–1.062; *p* = 0.019) (Figure 7D). Next, we assigned all patients into different subgroups according to age, gender, grade, and tumor stage to test if the prediction ability of the risk model could be applied to patients with different clinicopathologic characteristics. As expected, patients with high-risk had significantly worse overall survival than those with low-risk in all categories (Figures 8A–H). These results suggested that our risk model could serve as an independent prognostic indicator for HCC patients.

### Effects of the Glycolysis-Related Risk Model on Immune Cell Infiltration

**FIGURE 9:**
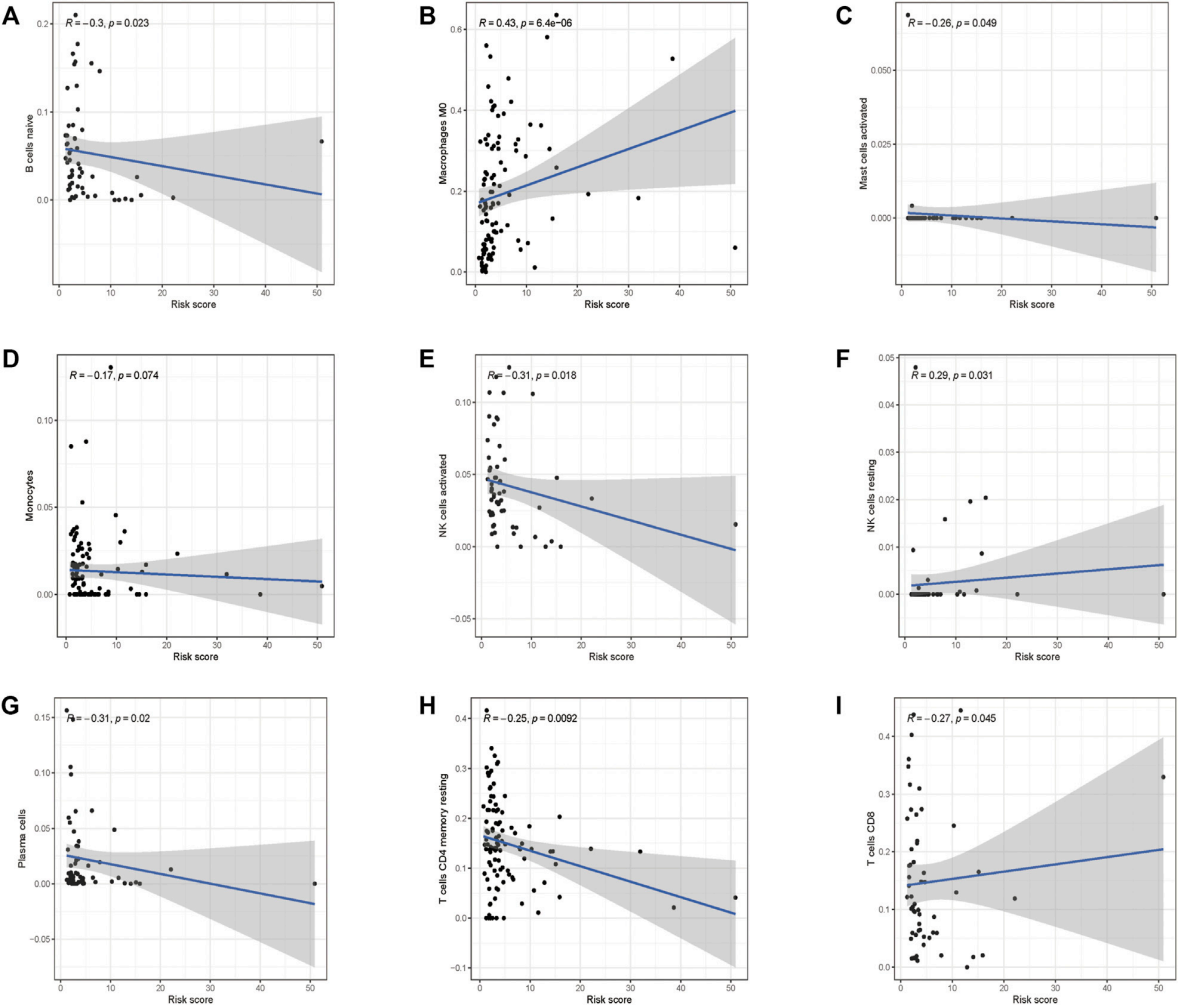
Correlations between the prognostic risk score and immune cell infiltration. **(A)** B cells, **(B)** M0 macrophages, **(C)** activated mast cells, **(D)** monocyte cells, **(E)** activated NK cells, **(F)** resting NK cells, **(G)** plasma cells, **(H)** resting CD4^+^ cells, and **(I)** CD8^+^ cells.

To investigate if the glycolysis-related gene signature influences immune cell infiltration, we further investigated the relationship between the risk score and immune cell infiltration. As shown in Figures 9A–I, higher abundance of B cells, mast cells, plasma cells, monocyte cells, activated NK cells, memory CD4^+^ T cells, and CD8^+^ T cells in the low-risk group was observed. Consistently, infiltration of M0 macrophages and resting NK cells was positively correlated with the risk score, which confirmed the correlation of the risk model and the immune microenvironment of HCC.

### Signal Pathways and Cellular Processes Related to the Prognostic Signature

**FIGURE 10:**
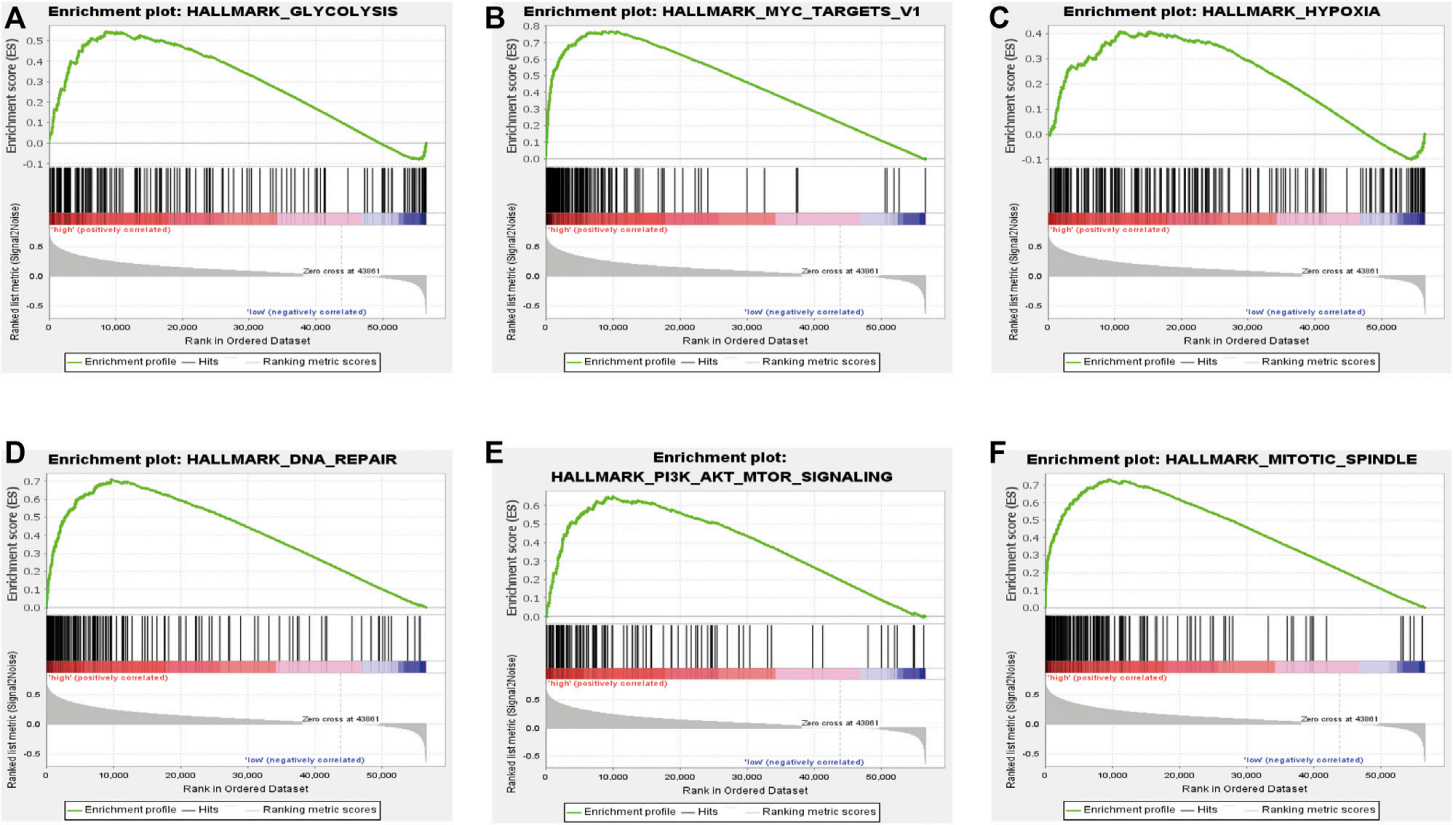
Enrichment plots of the Gene Ontology annotation from GSEA. GSEA analysis showing **(A)** glycolysis, **(B)** Myc-targets, **(C)** hypoxia, **(D)** DNA repair, and **(E)** PI3K-AKT-MTOR signaling. **(F)** Mitotic spindles were closely correlated with the high-risk phenotype.

To further study the effects of our prognostic risk model on biological function, we compared the signaling pathways differentially enriched between the high-risk and low-risk groups by performing GSEA. We found six biological processes/signal pathways were significantly activated in the high-risk group, including GLYCOLYSIS, MYC_TARGETS, HYPOXIA, DNA REPAIR, PI3K_AKT_MTOR, and MITOTIC_SPINDLE (Figures 10A–F), almost all of which were proved oncogenic previously.

### External Database Validation of the Prognostic Signature

**FIGURE 11:**
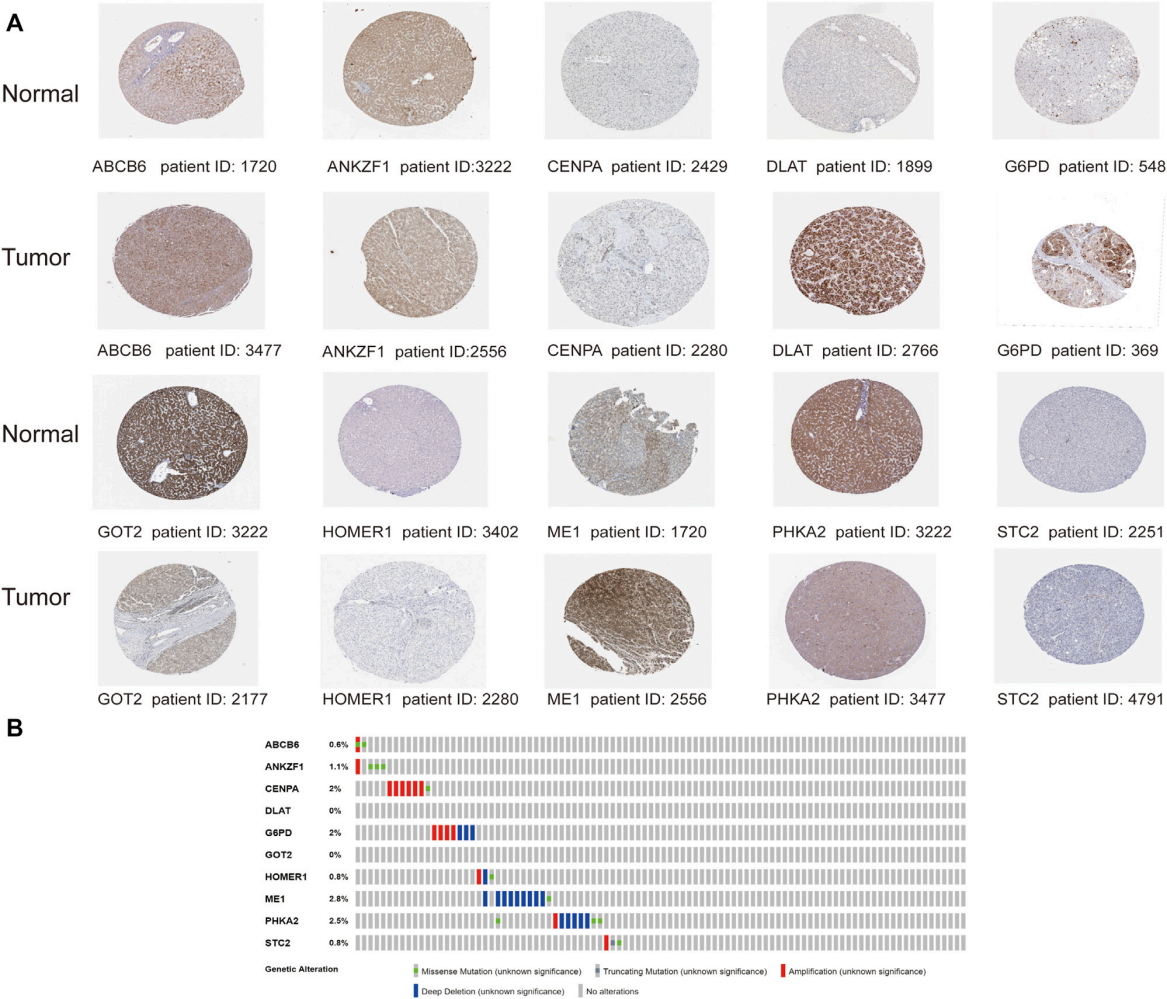
Analysis of the protein expression and gene mutation of the genes in the risk signature. **(A)** Immunohistochemistry staining data in HCC tumor tissues and normal tissues from The Human Protein Atlas. **(B)** Mutations of the 10 genes in HCC patients.

With the purpose of understanding genomic characteristics of the screened ten genes, their histological expression levels in HCC and normal tissues were obtained from the HPA database (Figure 11A). Differential protein expression of the ten genes was observed between normal and tumor samples. Among them, the protein expression of ABCB6, CENPA, DLAT, G6PD, HOMER1, and ME1 was obviously upregulated in HCC tissues but negligible difference in ANKZF1 and STC2 expression was observed. The expression levels of GOT2 and PHKA2 were higher in normal tissues. Moreover, we searched the cBioPortal database for mutation analysis, and mutation details of all the genes are shown in Figure 11B. The results indicated that *ME1* had the highest mutation rate (2.8%), while no mutation was detected in *DLAT* and *GOT2*.

### Knockdown of ME1 Inhibits Proliferation, Migration, and Invasion of Hepatocellular Carcinoma Cells

**FIGURE 12:**
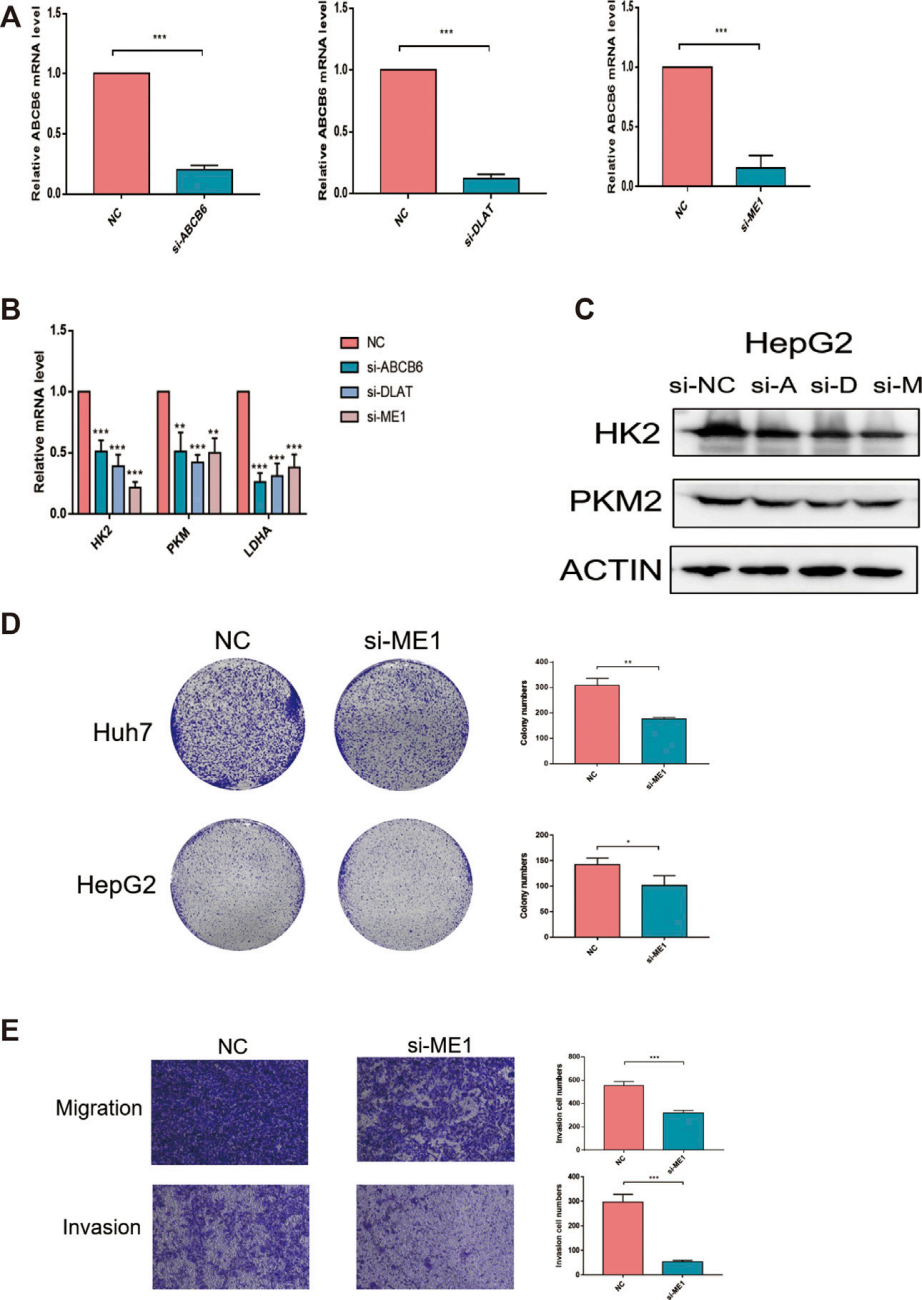
Knockdown of ME1 suppressed the malignant phenotype of hepatocellular carcinoma cells. **(A)** Knockdown efficiency of siRNA was assessed by qRT-PCR. **(B)** Relative mRNA expressions of HK2, PKM, and LDHA after knocking down ABCB6, DLAT, and ME1. **(C)** Protein expressions of HK2 and PKM2 after knocking down ABCB6, DLAT, and ME1. **(D)** Reduced colony numbers in ME1-silenced cells compared with control cells. **(E)** Different migration and invasion capacities between ME1-silenced cells and control cells. *p < 0.05, **p < 0.01, and ***p < 0.001. The data are presented as means ± SD of three independent experiments.

We chose genes that were used for risk model construction and whose protein expressions were significantly upregulated in HCC tissues for our experimental study. Since G6PD has been widely reported in previous research, we did not explore it here. We investigated that whether the expressions of ABCB6, DLAT, and ME1 could influence the key enzymes in glycolysis. As shown in Figures 12A,B, the mRNA expressions of HK2, PKM2, and LDHA were obviously suppressed after either ABCB6, DLAT, or ME1 was silenced. Consistently, decreased protein levels of HK2 and PKM2 were observed when one of the aforementioned three genes was knocked down (Figure 12C). Furthermore, we wondered if the malignant activities of hepatocellular carcinoma would be regulated by our screened genes. We found that genetic inhibition of ME1 significantly suppressed colony numbers, migration, and invasion of hepatocellular carcinoma cells (Figures 12D,E).

## Discussion

Reprogrammed energy metabolism is indispensable in the progression of human malignancy and has attracted increasing attention. The Warburg effect describes that tumor cells prefer aerobic glycolysis to support rapid biosynthesis even under normal oxygen conditions (Hanahan and Weinberg, 2011). Although aerobic glycolysis is inefficient to produce ATP compared with oxidative phosphorylation, several reasons can explain why aerobic glycolysis is able to meet the requirements of tumor growth and survival. First of all, a molecule of glucose only provides two molecules of NADPH and six molecules of carbon (Opdenakker et al., 1993), while sufficient NADPH supplied by the pentose phosphate pathway, a branch of glycolytic process, is conducive to cell proliferation and can protect cancer cells from oxidative stress (Pinthus et al., 2011). Second, proliferating tumor cells utilize glycolytic process to induce lactic acid secretion, and lactic acid helps to maintain cellular quiescence and protects tumor cells from drug killing (Yeh and Ramaswamy, 2015), which is one of the main causes of therapeutic resistance. Third, proliferating cancer cells require a continuous supply of macromolecules, and aerobic glycolysis is conducive to providing abundant necessary substrates for macromolecules in a hypoxic, low-sugar, and acidic tumor microenvironment (Shuch et al., 2013; Jiang et al., 2021). Finally, glycolysis enhances cancer cell proliferation with the help of a high ATP/ADP ratio (Christofk et al., 2008; DeBerardinis et al., 2008). The Warburg effect was first found in rat liver carcinoma in the 1920s, after then, growing evidence indicated that glycolysis could facilitate malignant transitions of HCC by producing the metabolites required for cell proliferation and migration (Feng et al., 2020). Targeting the glycolytic process may offer a therapeutic strategy to suppress HCC progression. Thus, we tried to identify prognostic biomarkers related to glycolysis during the treatment of patients with HCC.

In this regard, bioinformatic approaches were used to identify possible prognostic risk signature, we screened out 58 prognosis-related GRGs through univariate Cox analysis and divided HCC patients into two subtypes according to the GRGs expression. We found that cluster 2 affected immune cell infiltration of HCC and was closely associated with glycolytic process. We further screened ten genes to construct the prognostic risk model from 58 prognosis-related GRGs by performing LASSO regression analyses, and the risk model is valid to distinguish the HCC patients into high- and low-risk group in both the training and validation cohorts. And the risk score could distinctly distinguish overall survival despite clinicopathologic characteristics. Moreover, the risk score model for HCC patients could serve as an independent prognostic indicator.

The 10 prognostic risk genes identified in our study included *ABCB6*, *ANKZF1*, *CENPA*, *DLAT*, *G6PD*, *GOT2*, *HOMER1*, *ME1*, *PHKA2*, and *STC2*. Among these genes, the ABCB6 expression was upregulated in HCC, and its high expression was correlated with poor prognosis (Polireddy et al., 2011). Another bioinformatic analysis identified CENPA as one of the prognostic-related GRGs in clear cell renal cell carcinoma (Wu et al., 2020). Additionally, HCC tissues displayed higher expression of CENPA mRNA than adjacent normal tissues, and a low level of CENPA was correlated with better OS (Zhang et al., 2020). G6PD, an important rate-limiting enzyme of the pentose phosphate, was essential for tumor growth (Tang et al., 2015). GOT2 was proven to be related to the metabolism of tumor cells (Wang et al., 2021), low GOT2 expression was detected in liver tumor tissues, and its downregulation was correlated with worse prognosis (Liu et al., 2020). These findings were in agreement with our established risk model in this study. The ME1 expression could induce lactate production with reduced oxygen consumption (Liao et al., 2018); its high expression also indicated poor survival (Wen et al., 2015). Studies showed that STC2 transcript was upregulated in HCC tissues, and higher serum STC2 level was correlated with larger tumor size and shorter OS and DFS of HCC patients (Wang et al., 2012; Zhang et al., 2014). Moreover, STC2 could promote glycolytic process in nasopharyngeal carcinoma (Li et al., 2021). Another study identified *ANKZF1* and *DLAT* as glycolysis-related genes to predict the survival in colon adenocarcinoma patients (Chen et al., 2020). However, the significance of HOMER1 and PHKA2 in glycolysis and in evaluating the prognosis of hepatocellular carcinoma patients remains unknown, and our current study suggested a possible role of them in glycolysis and HCC progression.

HCC is inflammation-associated, and its immune microenvironment reflects the immune infiltration. An immune-suppressive TME supports tumor progression and metastasis *via* building a symbiotic relationship with tumor cells (Fu et al., 2019). The influence of the tumor immune microenvironment by the glycolytic process still remains undefined; our analysis revealed that the infiltration of B cells, mast cells, monocytes, plasma cells, activated NK cells, memory CD4^+^ T cells, and CD8^+^ T cells was significantly increased in the low-risk group, while resting NK cell infiltration was positively correlated with the risk score. B cells are regarded as the main effector cells in humoral immune response; it could promote tumor recession through secreting cytokines, enhancing cytotoxic T cell response to directly kill cancer cells (Tokunaga et al., 2019). Monocytes show dichotomous roles in tumor development depending on its plasticity in response to environmental stimuli; they could induce immune tolerance or active antigen-presenting cells under specified conditions (Ugel et al., 2021). Plasma cells are recognized as end-stage products of B-cell differentiation, which both provide humoral immunity and store immunological memory (Radbruch et al., 2006). Interestingly, mast cells possess dual roles in tumor progression, and they have the potential to induce tumor angiogenesis, metastasis, and invasion; on the contrary, they are also capable of shaping adaptive immune responses to tumors (Marichal et al., 2013). NK cells are innate lymphocytes that are responsible for immune surveillance and clearance of multiple nearby cells expressing malignant transformation-related surface markers (Shimasaki et al., 2020). Memory CD4^+^ T cells are likely to control and maintain protective immune responses (Stockinger et al., 2006). CD8^+^ T cells could recognize and kill cancer cells directly, which are the core ingredients for anti-tumor immunity (Iwahori, 2020). The immune cell infiltration landscape indicated that the glycolysis-related risk model could reflect the tumor immune microenvironment; the low-risk group showed favorable immune infiltrates, which is consistent with better overall survival.

Admittedly, there are several limitations to our study. First, although we verified the prognostic risk model using half of the samples in TCGA HCC cohort and in another independent HCC cohort (GSE14520), a large-scale multicenter cohort would allow for stronger validation. Second, further clinical and *in vivo* experimental evidence is required to unravel the molecular mechanisms of prognostic significance of our risk predictive model. Third, the development and progression of HCC are influenced by many other factors, so it may have an inherent defect by using gene expression data to construct a predictive model.

## Data Availability

The raw data supporting the conclusion of this article will be made available by the authors, without undue reservation.
