# Supplementary material for: Effects of Glycolysis-Related Genes on Prognosis and the Tumor Microenvironment of Hepatocellular Carcinoma
Source: Front Pharmacol. 2022 Jul 18;13:895608. doi: 10.3389/fphar.2022.895608 (PMC9340275; doi:10.3389/fphar.2022.895608)
Supplement: Supplementary file 1 [file DataSheet1.PDF]

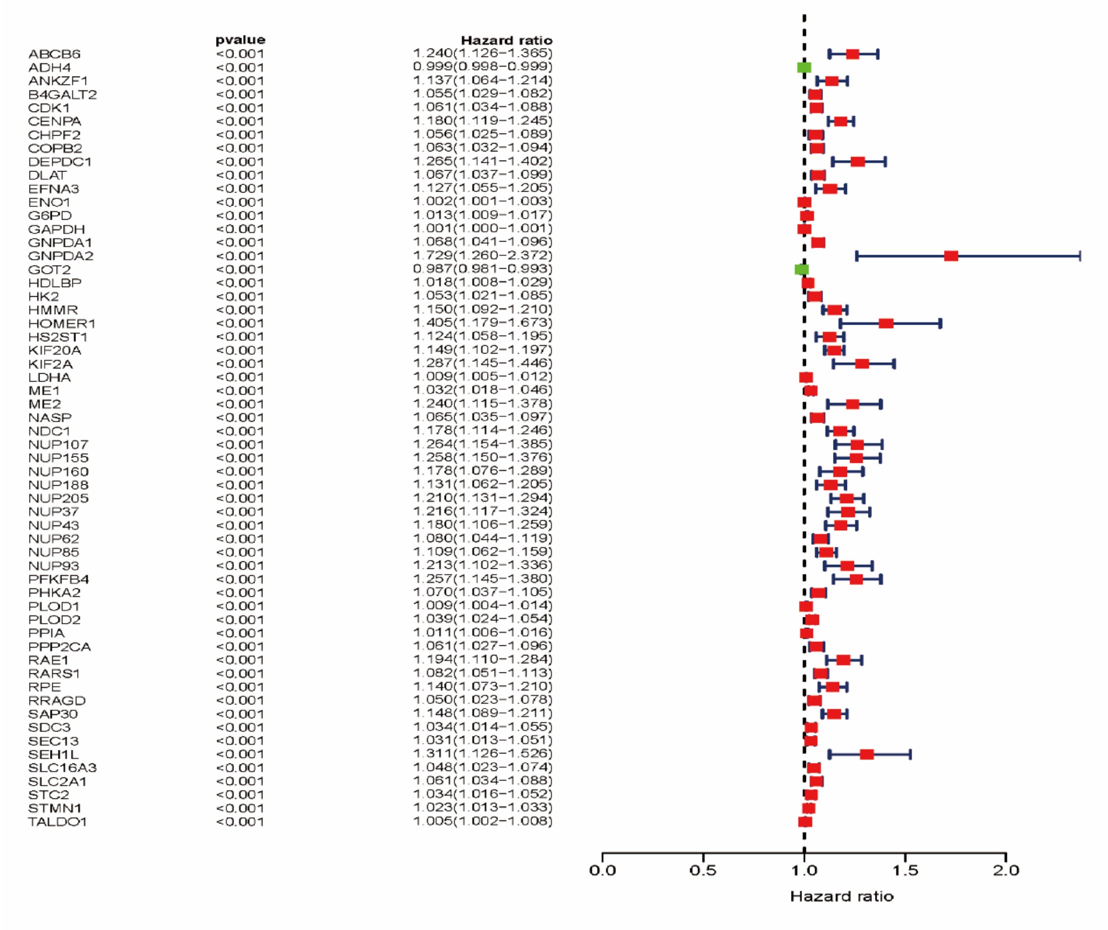

Figure S1 Heatmap showing the differential expression profiles of 292 glycolysis-related genes.

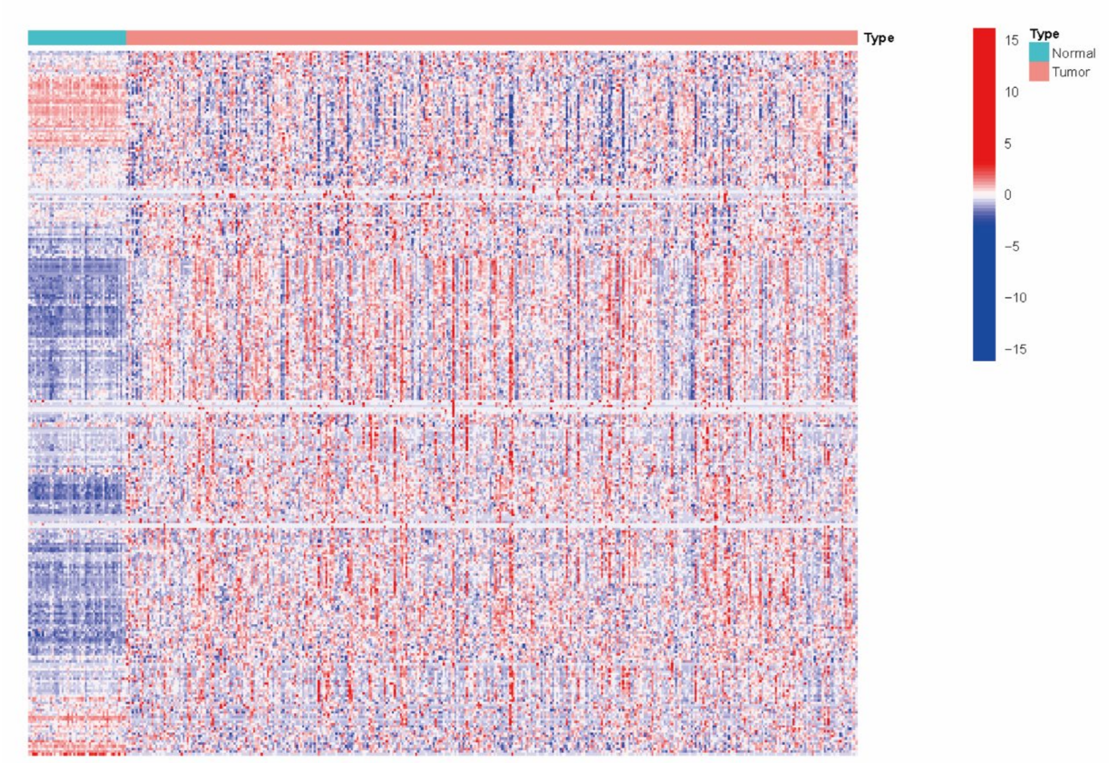

Figure S2 Univariate cox regression screened the prognostic-related GRGs. The forest plot shows hazard ratios of each gene.

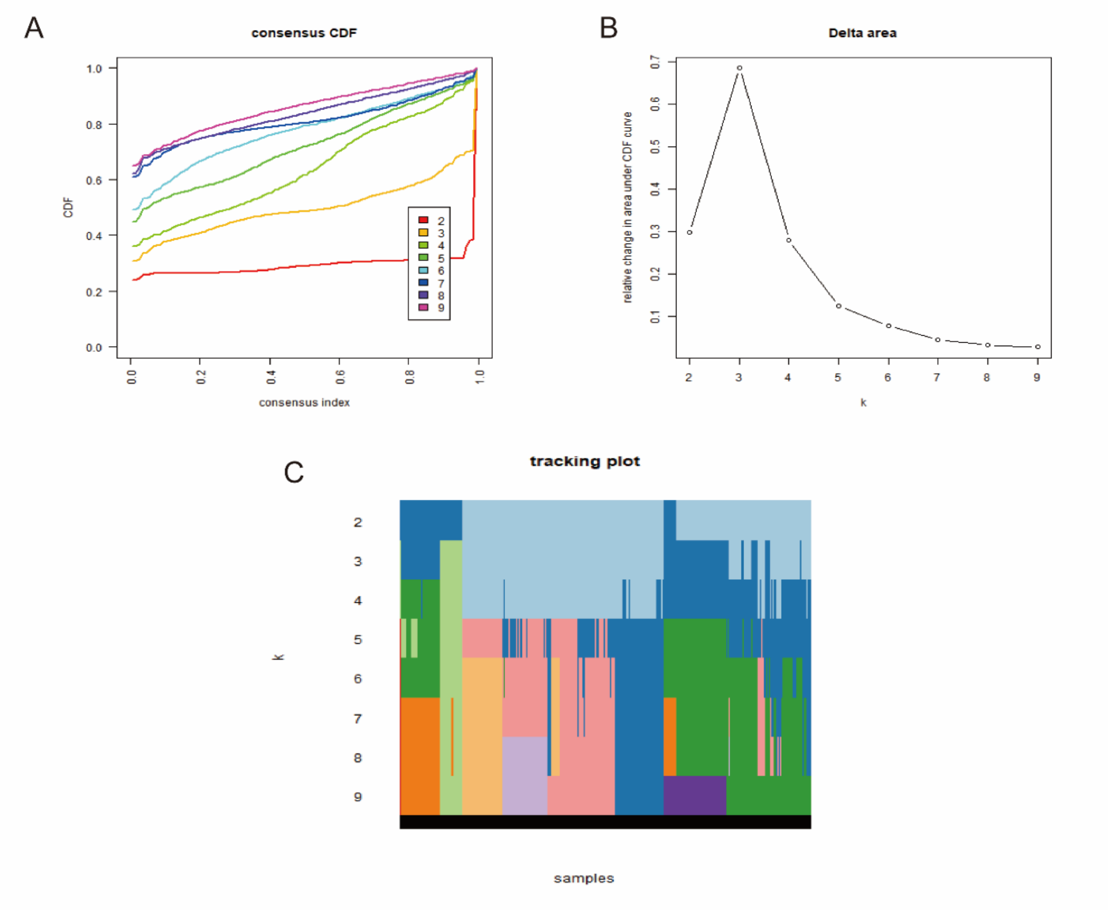

Figure S3 Consensus clusters by prognostic-related GRGs in TCGA cohort. (A) Consensus clustering cumulative distribution function (CDF) for k=2 to 9. (B) Relative change in area under the CDF curve (k=2 to 9). (C) Tracking plot (k=2 to 9).

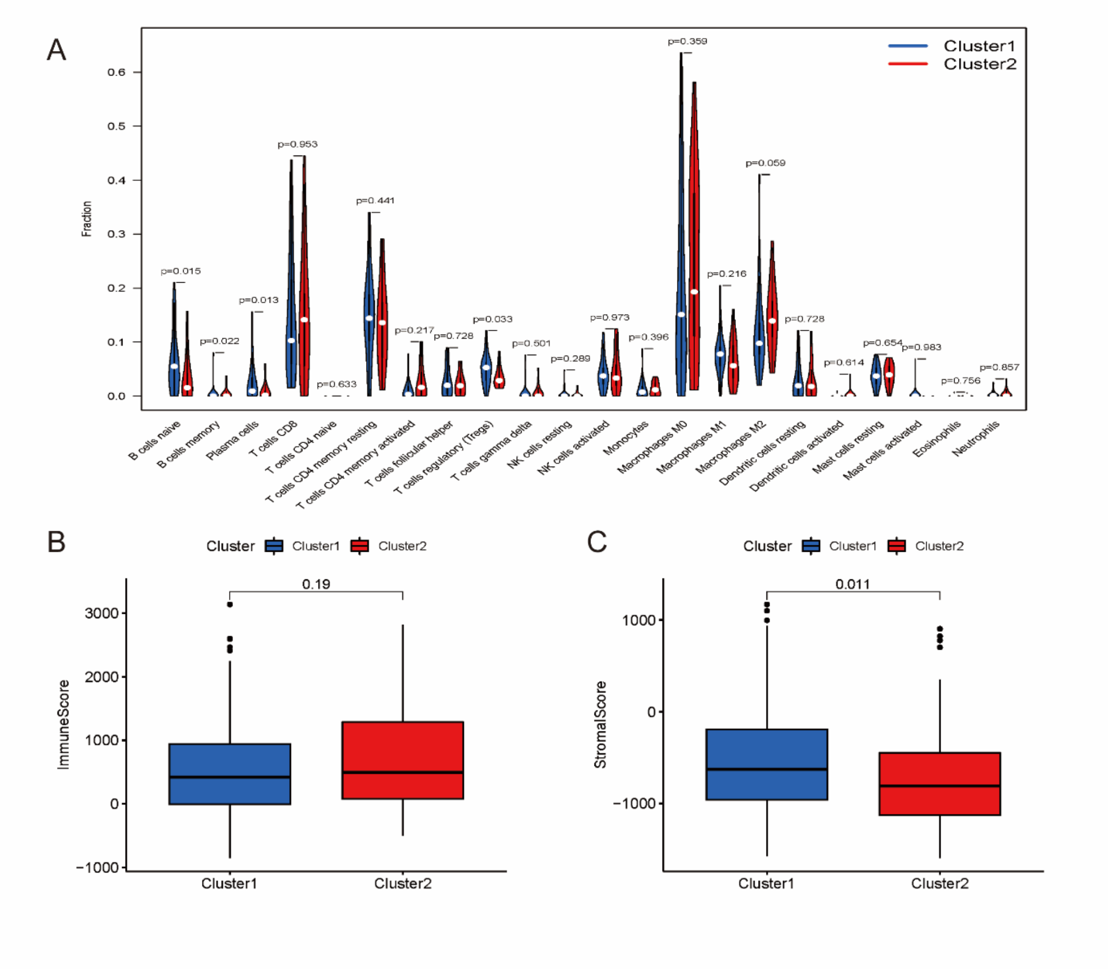

Figure S4 Immune cell infiltration and TME score in two clusters in TCGA cohort.(A) The infiltration of immune cells in two subtypes. *$p < 0.05$. (B) Immune score in the cluser1/2 subtypes. (C) Stroma score in the cluser1/2 subtypes.

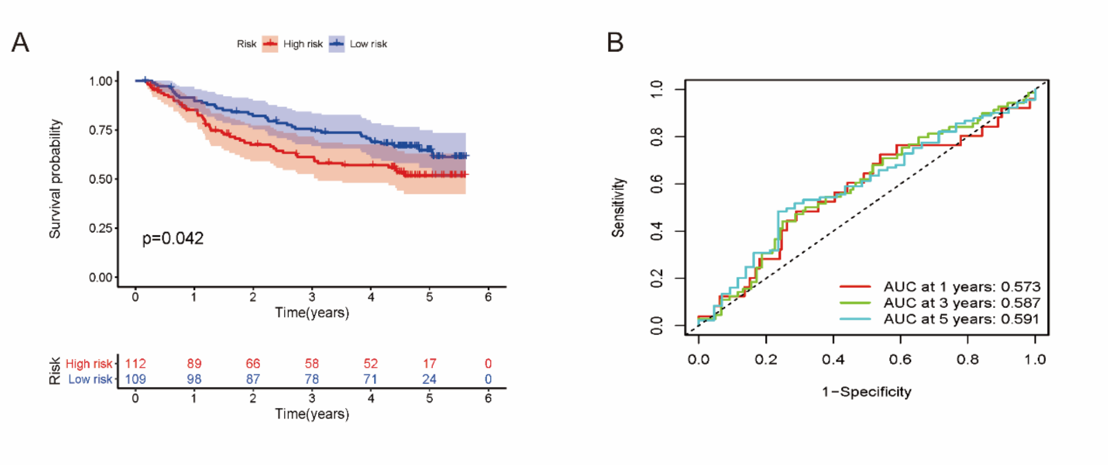

Figure S5 Validation of the prognostic signature in GSE14520 dataset. (A) The survival analysis between high risk group and low risk group. (B). ROC curve showing the prediction accuracy of the prognostic signature.
